# Supplementary material for: DeepD3, an open framework for automated quantification of dendritic spines
Source: PLoS Comput Biol. 2024 Feb 29;20(2):e1011774. doi: 10.1371/journal.pcbi.1011774 (PMC10903918; doi:10.1371/journal.pcbi.1011774)
Supplement: S1 Supporting Information — (PDF) [file pcbi.1011774.s001.pdf]

DeepD3, an Open Framework for Automated Quantification of Dendritic Spines

Martin H P Fernholz, Drago A Guggiana Nilo, Tobias Bonhoeffer, and Andreas M Kist

Supplementary Information
Figures and Tables

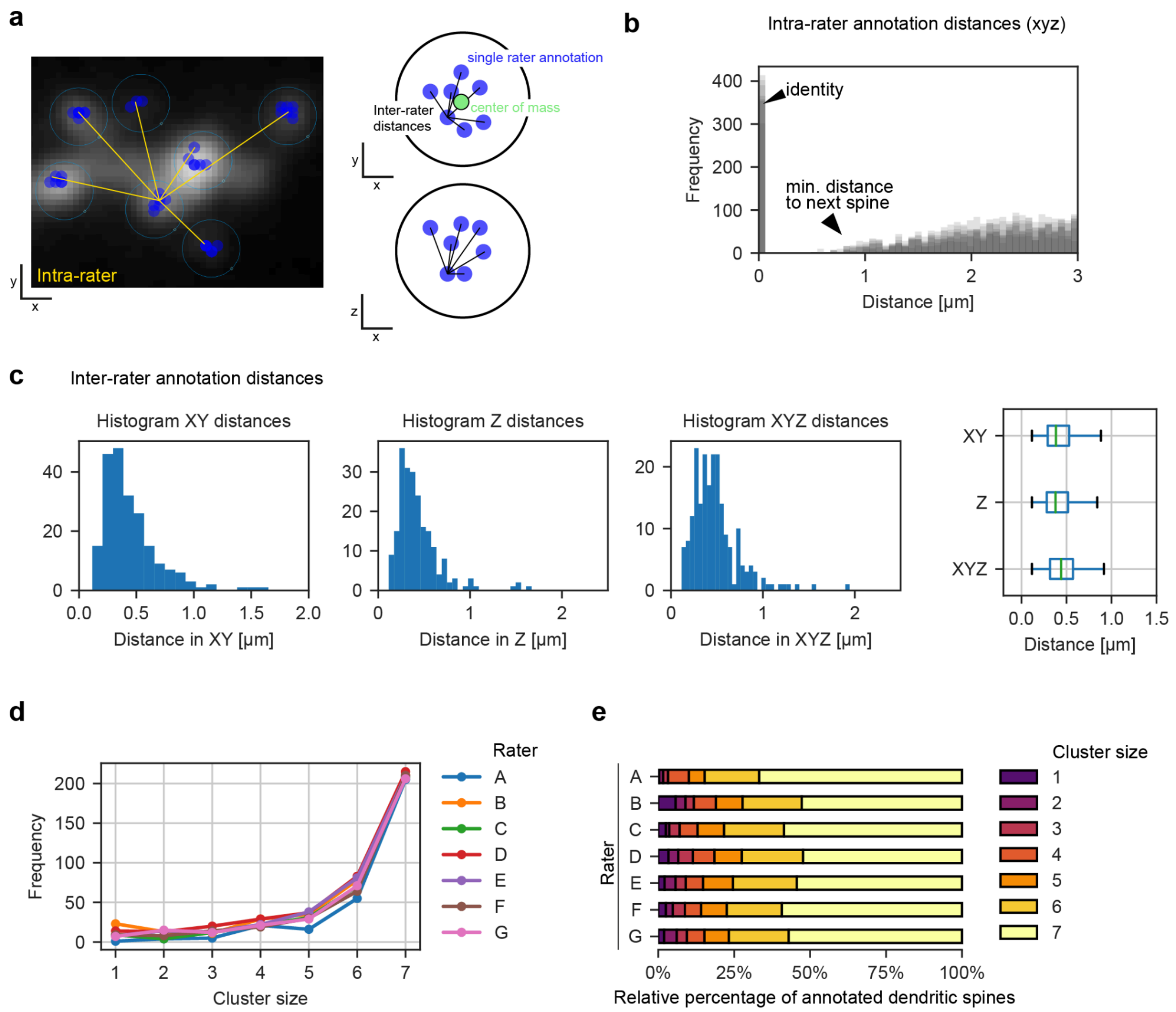

Figure A. Inter- and intrarater analysis. a, Comparison of intra- and inter-rater analyses. Left, an ROI in the benchmarking dataset with spine annotations (blue points) of 7 human experts. Distances between points of a single rater (intra-rater) are displayed in yellow. Right, distances between raters (inter-rater) are compared for a single dendritic spine in xy (top) and xz view (bottom). **b**, Three-dimensional Euclidean distance across annotated dendritic spines, overlaid across raters. Identity signifies the same dendritic spine (i.e. distance of 0). Minimal distance to the next spine starts at a Euclidean 3D distance of approximately 0.6 µm **c**, Inter-rater annotation distances for manually matched dendritic spines in µm. **d**, Frequency of found dendritic spines across raters A-G depending on the cluster size. **e**, Relative distribution of annotated dendritic spines per rater across cluster sizes. The majority of all annotated spines (> 50%) per rater are also identified by all other raters.

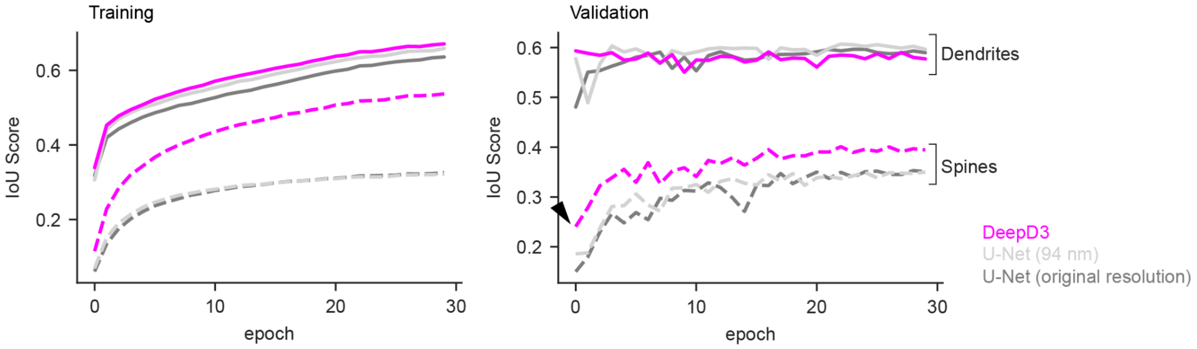

Figure B. DeepD3 architecture outperforms U-Net. The Intersection over Union (IoU) score shows the segmentation quality of the deep neural network compared to the ground truth. Performance comparison of a strong baseline (U-Net, training dataset in original resolution (dark gray) or fixed resolution (light gray)) compared to our proposed DeepD3 architecture (magenta) on the training (left) and validation (right) dataset across training epochs. NB: the DeepD3 dendritic spine performance is constantly above the U-Net baseline in the validation dataset. In addition, it converges faster and already shows superior performance after the first training epoch (black arrowhead).

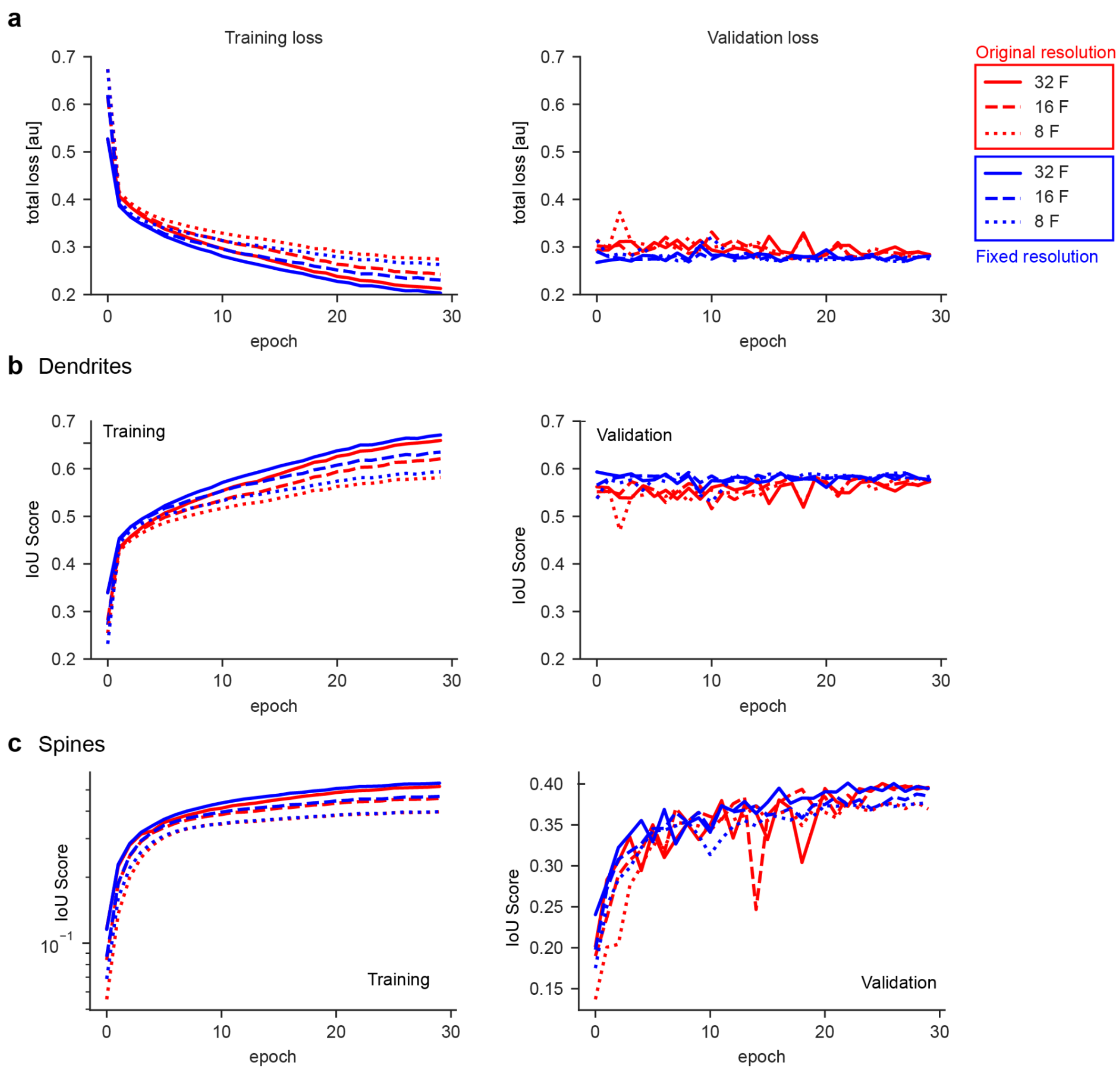

Figure C. DeepD3 learns to detect dendrites and dendritic spines.
a, Combined training loss for dendrites and spines (see Methods in the main manuscript). Shown are different DeepD3 scaling variants with different line styles (8F for dotted, 16 for dashed, and 32F for solid lines) for either a fixed training resolution (here 94 nm, shown in blue) or utilizing the original (mixed) training data resolutions (red). **b**, Intersection over union score (IoU) across training epochs for training data (top panel) and validation data (bottom panel). **c**, IoU score across training epochs for spines. N.B. spine IOU score converges between epochs 20-30.

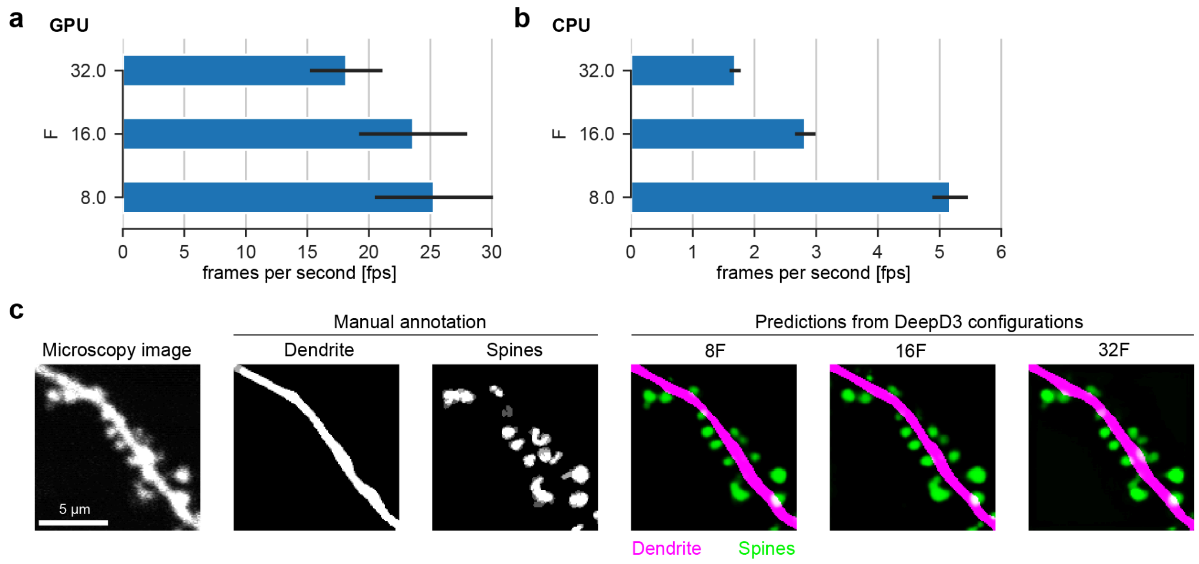

Figure D. DeepD3 neural architecture allows scaling to enhance inference speed by minimally sacrificing prediction quality.

a, Inference speed of a single 512x512 px tile measured in frames per second on a consumer GPU (NVIDIA RTX A4000) across DeepD3 scaling variants, denoted as 8F, 16F, and 32F (set filters in f_{base}). **b**, Inference speed of a single 512x512 px tile measured on a consumer CPU (AMD Ryzen 3950X) across DeepD3 scaling variants. **c**, Exemplary data tiles showing together with manual annotation and their respective network prediction (dendrite in magenta, spines in green) across DeepD3 scaling variants.

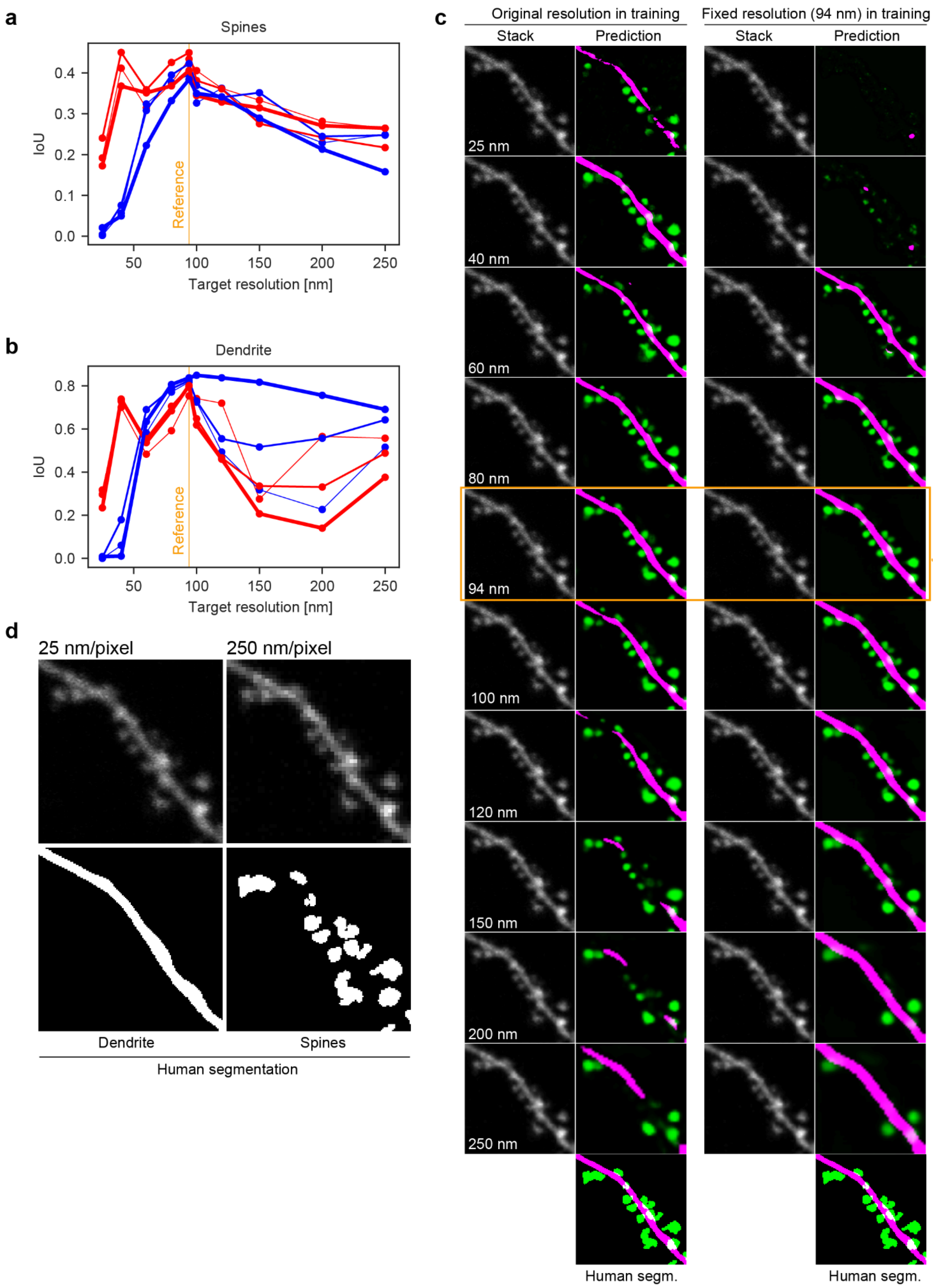

Figure E. DeepD3 is stable across resolutions depending on the training paradigm.
a, Intersection over Union (IoU) score for spine predictions across artificially generated resolutions from the validation dataset. Original (mixed) resolution in red, fixed resolution to 94 nm in blue. Line thickness represents used base filters (8, 16 or 32, for light, medium, or thick line strokes, respectively). **b**, Same as panel a but for dendrite predictions. **c**, Left column: Example tile of raw data of the validation dataset across resolutions. Second column DeepD3 prediction using a neural net trained on mixed resolution on raw data of various resolutions (dendrite prediction in magenta, spines prediction in green). Third column: same as left column. Fourth column: same as the second column but for a neural net trained on a fixed resolution. The bottom row shows the pixel-precise segmentation provided by a single human expert.. **d**, Two example tiles to showcase the effect of resizing a given tile to different resolutions. Top: scaled raw data. Bottom: human-segmented dendrite (left) and spine (right) segmentations of the raw data shown on top.

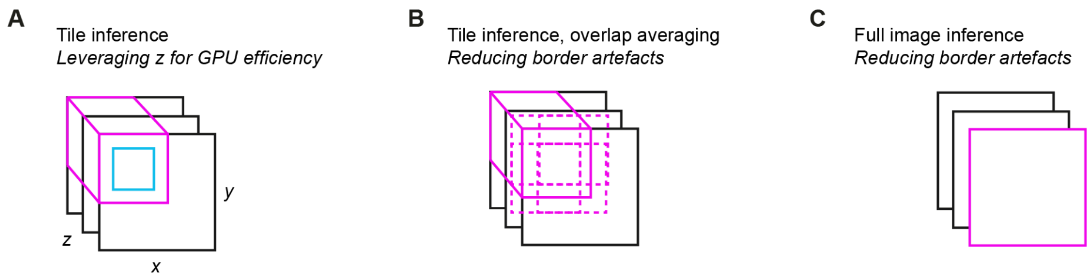

Figure F. Available inference modes for the DeepD3 framework.
a, Inference of image tiles (pink) that are slid in x and y. Only a smaller inset of the tile (blue rectangle) is used for the final prediction. To leverage the full GPU power, we utilize the z-depth to avoid IO-bound bottlenecks (see Online Methods). **b**, Inference of image tiles (pink) but overlapping in four directions and averaged such that border artifacts are reduced. **c**, Full image inference at a given z-depth.

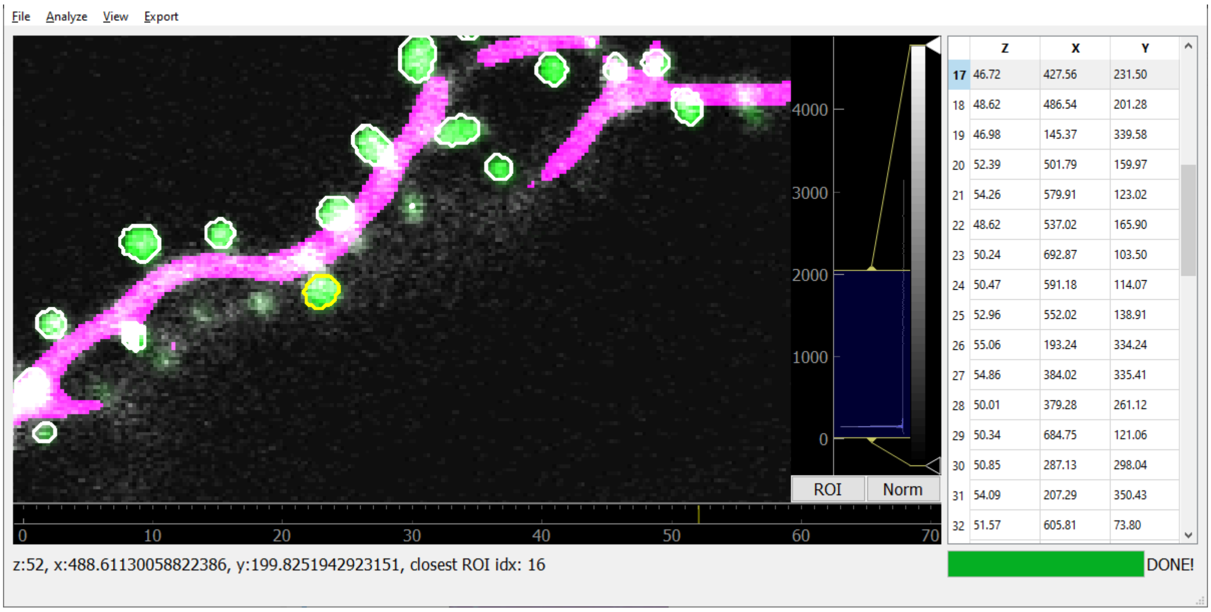

Figure G. Graphical User Interface.

Example screenshot of the DeepD3 Graphical User Interface (GUI). Tools can be selected via a drop-down menu (top). In the center part of the GUI, the loaded raw data (grayscale), performed predictions of dendritic spines (green) and dendrites (magenta), as well as generated spine ROIs (white or yellow outlines of dendritic spine predictions) are displayed. To the right of the main window, the contrast of the raw data can be adjusted (grayscale bar). Identified spine ROIs and their location in three dimensions are listed per z-level on the right side of the GUI (table with Z, X, Y columns and numbers as rows). Below is a loading bar (green) that communicates the progress of the current processing step to the user.

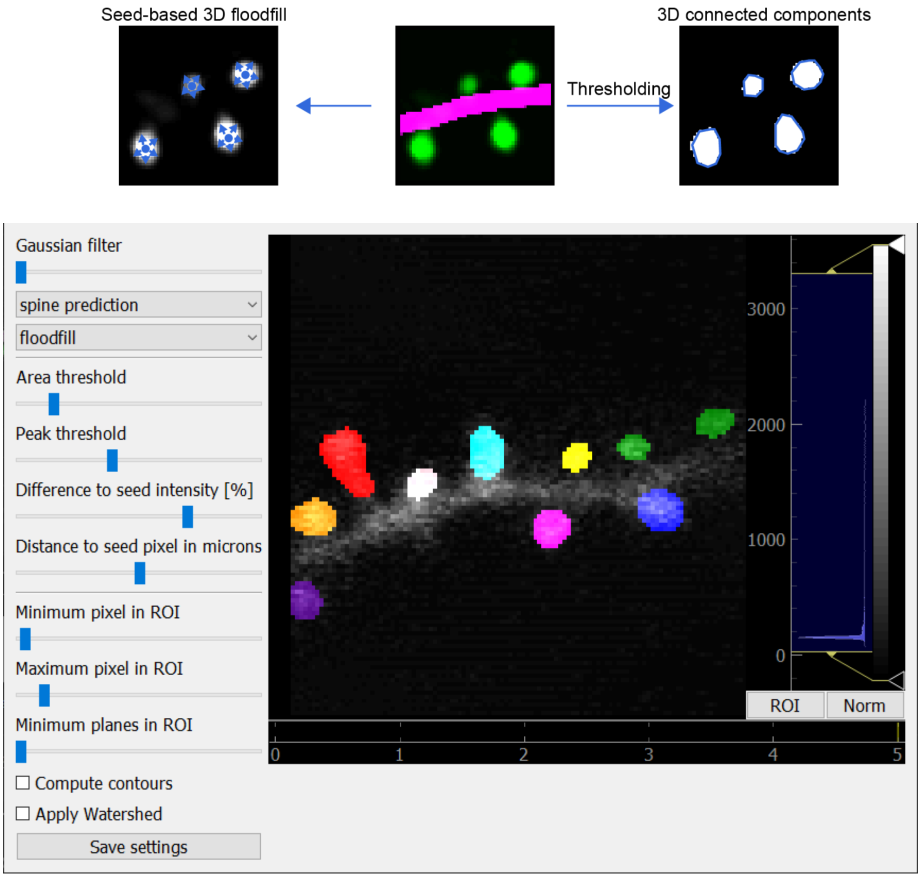

Figure H. Testing 3D ROI building in Graphical User Interface.
Top: two approaches of building spine ROIs from the cleaned spine prediction map (seed-based 3D flood-fill and 3D connected components). Bottom: DeepD3 built-in option to generate real-time feedback when building 3D ROIs to rapidly fine-tune user-defined hyperparameters. On the right side of this GUI, hyperparameters can be defined, and the main window shows the resulting spine ROIs (in different colors). Raw data are shown in grayscale and can be contrast adjusted (histogram on the right).

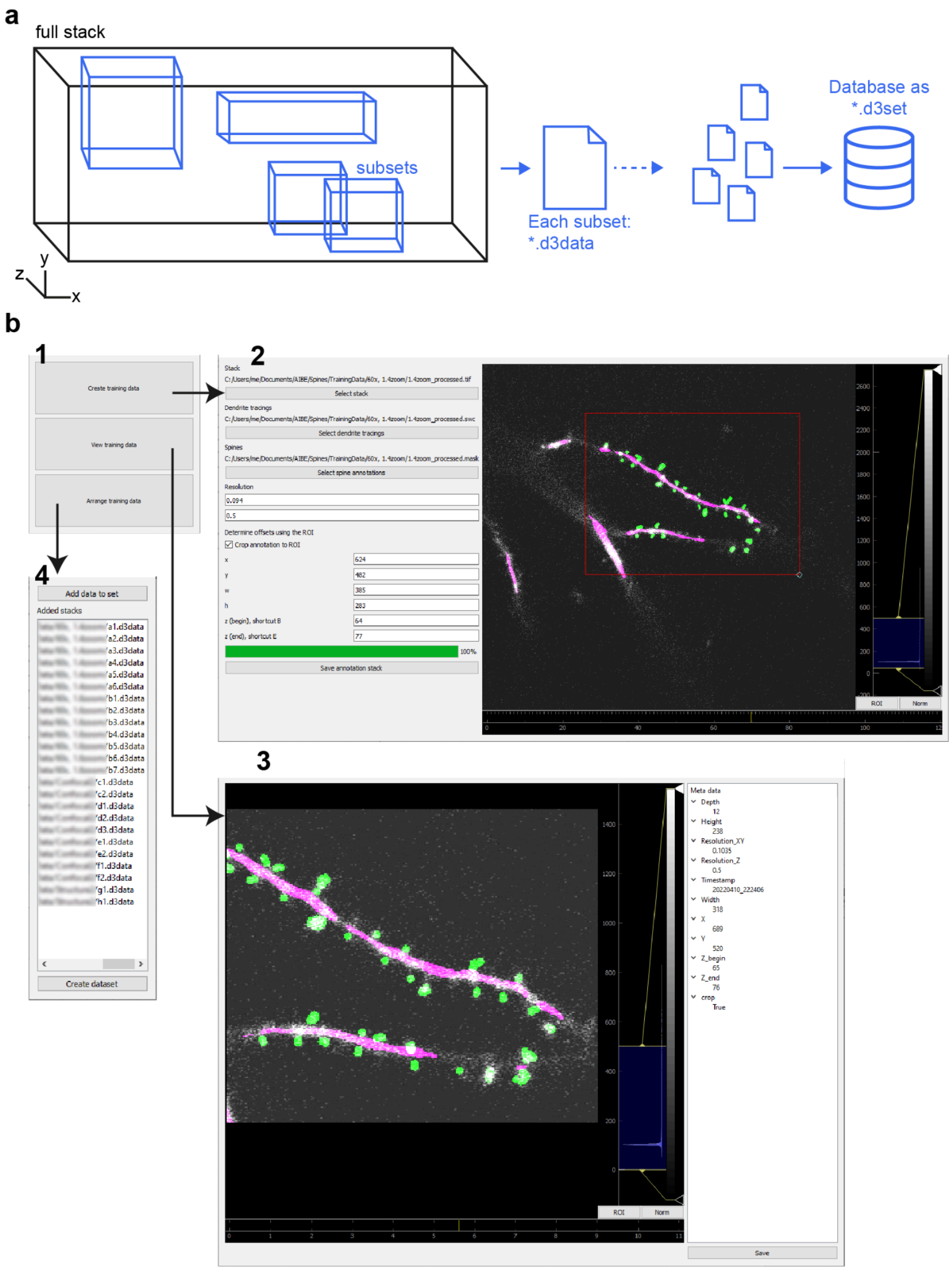

Figure I. Graphical User Interface for Training Data Compilation.
a, Workflow of the training data compilation pipeline. A given (partially) annotated stack (black box) can be used as training/validation/test data source. One can select one or multiple subsets (in x,y,z) each of which is saved as a *.d3data file. User-selected files can be compiled to a database-like *.d3set that contains these individual subsets. The *.d3set files can be further used in the training pipeline. **b**, Graphical User Interface elements that conveniently address the workflow in panel a. (1) is the main window, where one can access the subsequent features. (2) allows stack loading, dendrite reconstruction and subset generation, as well as *.d3data saving. (3) allows revisiting saved *.d3data subsets. (4) compiles selected files to a *.d3set.

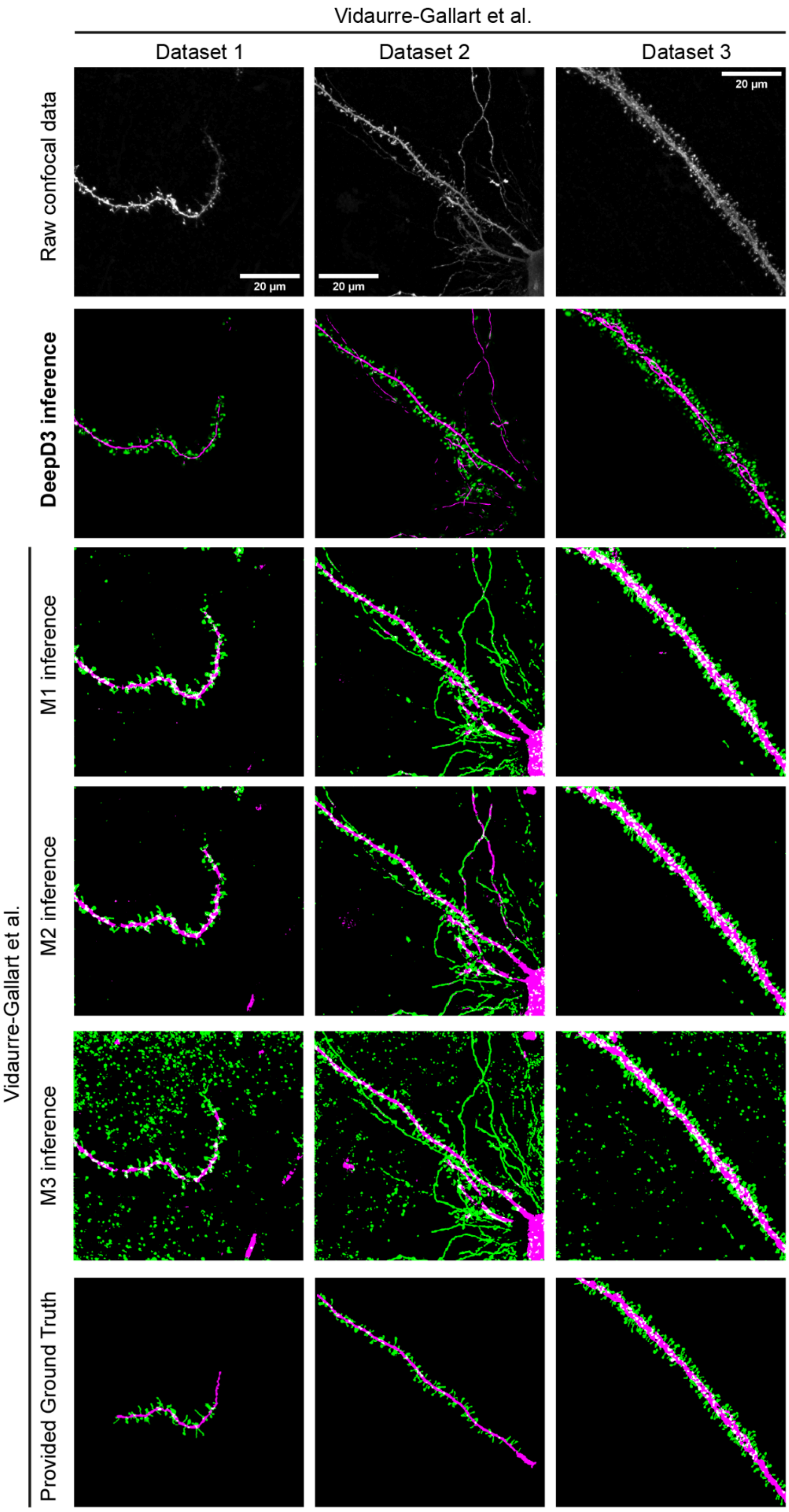

Figure J. Qualitative evaluation of contemporary methodology.
Top row: original raw TIFF stack datasets kindly provided by Vidaurre-Gallart et al. (2022). Second row: qualitative DeepD3 prediction performance on the raw data. Each plane-by-plane inference of the raw TIFF stack was performed using the DeepD3-32F network trained on unconstrained pixel resolution. Raw predictions were cleaned using the default DeepD3 cleaning settings and procedures. Third to fifth row: inference using the networks M1, M2 and M3, respectively, kindly provided by Vidaurre-Gallart et al (2022). Bottom row: ground-truth labels kindly provided by Vidaurre-Gallart et al (2022).

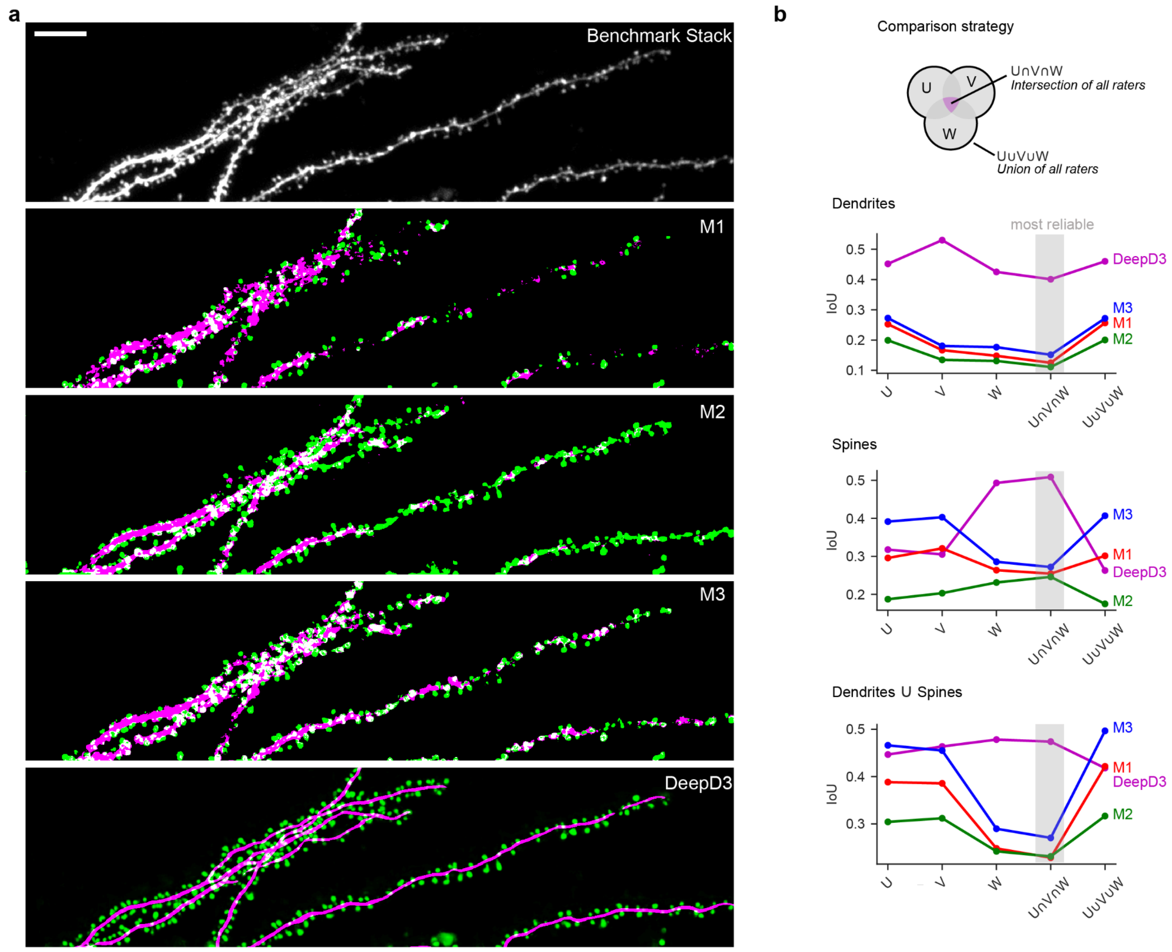

Figure K. Qualitative evaluation of contemporary methodology.

a, Original raw TIFF stack from the DeepD3 benchmark dataset (top) was analyzed using the methodology described in Vidaurre-Gallart et al. (2022, center three images). The DeepD3 prediction (bottom) was generated using the DeepD3-32F network trained on unconstrained pixel resolution. Raw predictions were cleaned using the default DeepD3 cleaning settings and procedures. Qualitatively, the approach presented by Vidaurre-Gallart et al., (2022) fails to accurately segment dendrites and dendritic spines in the DeepD3 benchmark dataset. Scale bar is 10 µm. **b,** Quantification of segmentation performance across individual raters, their intersection and their union annotation (see schematic on top). The IoU score is shown across neural networks M1, M2 and M3 (Vidaurre-Gallart et al., 2022) colored in red, green and blue, respectively, and DeepD3 (magenta). The intersection of all raters is thought as most reliable (indicated with shaded box). Performance is shown for spines, dendrites and the union of dendrite and spine labels.

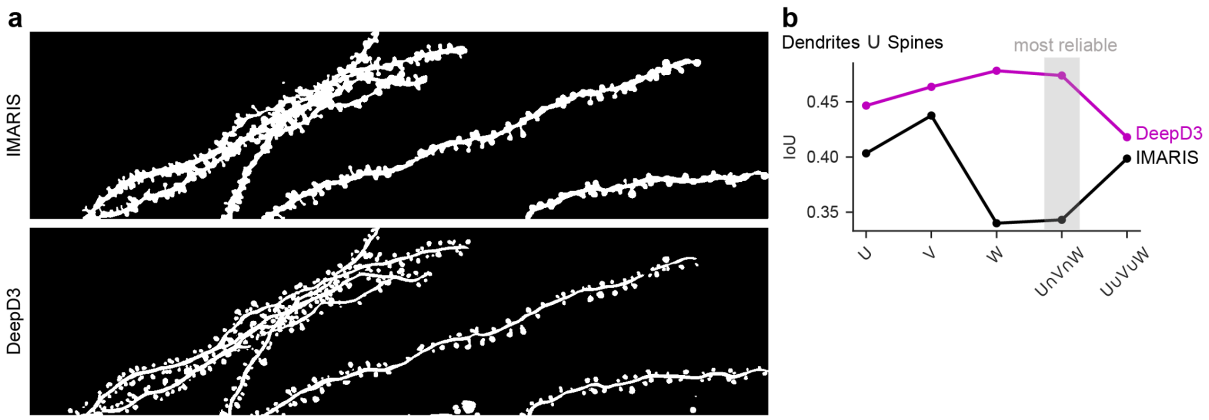

Figure L: DeepD3 qualitatively and quantitatively outperforms semi-automatic state-of-the-art methods. **a,** Maximum intensity z-projection of the benchmark dataset with dendrites/spines extraction using IMARIS (upper panel) and DeepD3 (lower panel, union of dendrite and spine prediction with a threshold of 0.5). **b,** IoU quantification to human segmentations as introduced in Extended Data Figure 12b.

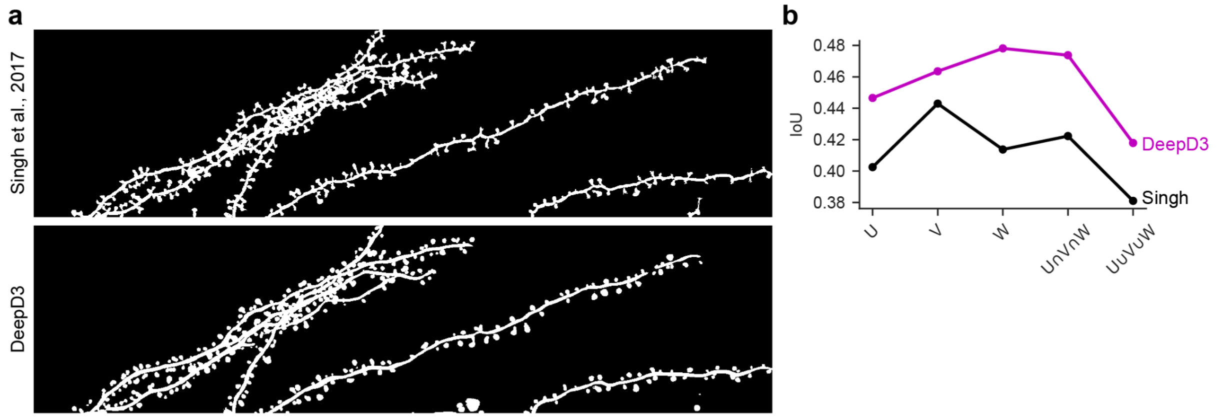

Figure M: DeepD3 qualitatively and quantitatively outperforms fully automatic state-of-the-art methods.
a, Maximum intensity z-projection of the benchmark dataset with dendrites/spines extraction using the method introduced by Singh et al., 2017 [10] (upper panel) and DeepD3 (lower panel, union of dendrite and spine prediction with a threshold of 0.5). **b**, IoU quantification to human segmentations as introduced in Extended Data Figure 12b.

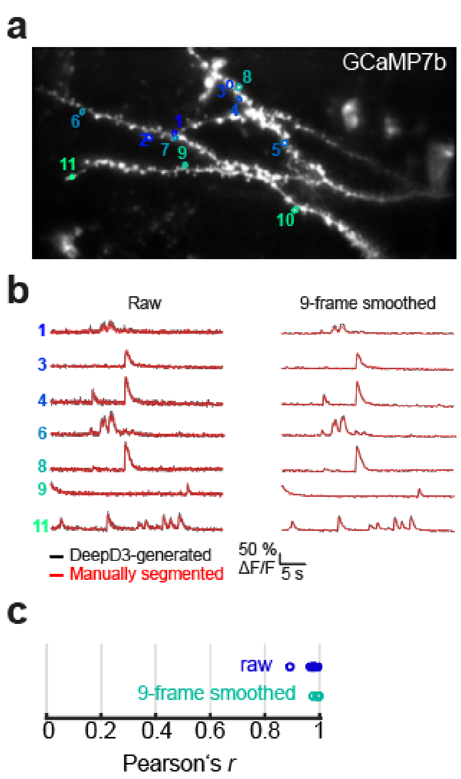

Figure N. DeepD3-generated and manually segmented spine ROIs generate near-identical timecourse calcium fluctuation data.

a, Average projection of the analyzed calcium-imaging movie with DeepD3-generated spine ROI outlines in color and assigned numbers. **b**, Raw and 9-frame smoothed (left and right, respectively), calcium transients ($\Delta F/F_0$) of responsive spines extracted using DeepD3-generated (black) and manually segmented ROIs (red). **c**, Pearsons's correlation coefficient *r* raw (blue) and 9-frame smoothed (turquoise) traces shown in b.

Reference	Software Name / Acronym	Brief description of spine detection	Dimensionality of input data	Notes	Code Availability
Ekaterina et al., 2023 [1]	-	Image binarization, conversion to polygonal mesh via Poisson surface reconstruction, dendrite skeletonization via Mean Curvature Skeleton algorithm, spine segmentation via vertex of skeleton, spines morphology is extracted via polygonal meshes, spine parameters are extracted (including novel chord length distribution histogram), subsequently classified via SVM, and finally clustered.	2D	Unclear whether it works in images containing multiple disconnected dendritic stretches. Main purpose is to classify spine morphology in high quality image data, not detect spines. Required image quality seems to be high, as only a subset of spines could be detected in a lower quality public dataset (see S3).	Analysis code is available. Dendritic spine meshes are available, raw data is not available.
⚫ Vogel et al., 2023 [2]	-	Faster R-CNN with feature extractor ResNeXt-101 pretrained on MS COCO dataset and refined via transfer learning with own spine dataset (training data). The network identifies bounding boxes (regions; via region proposal network), which get subselected using a box classifier (BoxC) and are subsequently assigned a likelihood score based on the network's confidence on the box containing a dendritic spine. Spine necks are computed using multi-stencil fast marching method of the spine head center and identify the shortest 3D distance to a nearby dendrite. Then, additional constraints (smoothness and complexity) are applied to identify the actual spine neck and not only the shortest path length. Segmentations are then used to calculate volume and FWHM estimates.	3D	This network does not perform segmentation. It only identifies regions (boxes) which contain spines. Consequently, spine counting is possible, however, spine-shaped analyses, etc.. are not. Test dataset is annotated by 5 expert raters.	Analysis code is available. Data is not available.
Argunşah et al., 2022 [3]	SpineS	Use is intended for time-series data. Data is registered globally (for each time point) then locally (for each spine). Dendrite is segmented (2D-median filter + Otso thresholding) and	3D + time	SpineS seems to underestimate spine counts in simulated data (Fig. 8).	Data is available. Only an old version of the code is available.

		medial axis computed (fast marching distance transform). Connected spines are then removed. Instead, spines are detected using speeded-up robust features (SURF) and a CNN, which set the seed points for subsequent watershed segmentation and additional shape modification.			
⚫ Vidaurre-Gallart et al., 2022 [4]	DeepSpineTool / DeepSpineNet	3D convolutional neural network with model options, watershed segmentation.	3D	Second use of deep learning in spine detection. Data available upon request. Allows for manual post-editing.	Yes
Das et al., 2021 [5]	3dSpAn	Preprocessing: 3D median filter, bilinear interpolation to scale image. Segmentation: semi-automated thresholding, seed selection, and segmentation. From individual spine ROIs, morphological features are extracted (e.g. volume or head width).	3D	The authors provide several resources (website, guide, sample data, source code, installer options).	Yes
Levet et al., 2020 [6]	SpineJ	Manual wavelet-based filtering to binarize the image. Reconnect spine heads to dendrite in segmented image via gradient field computations. The segmented image is then skeletonized via polygons, splines are calculated and used as seeds to detect spines via Delaunay triangulation. Finally, spine features (e.g. neck length) are quantified.	3D	Works as a ImageJ plugin. Designed for super-resolution images.	Yes
Rada et al., 2018 [7]	- -	Preprocessing: 2D Gaussian filter followed by dot enhancement of input images is performed. Spines are then detected via so-called SIFT method, which is based on a support vector machine (SVM) that classifies image feature locations. These locations are then used in watershed-variational based segmentation to detect spines. Registration across time: SIFT-based landmark locations are used to rigidly register images across time points. Spines are mainly matched based on	3D + time	Example data also available. Performed comparison to NeuronIQ. Employed two expert raters but only reported their average performance, no direct comparisons between the two.	Yes

		Euclidean distance criteria of landmark locations.			
⚫⚫<br>Xiao et al., 2018 [8]	-	Deconvolution during preprocessing, convolutional neural networks.	2D	First use of deep learning in spine detection.	No
Smirnov et al., 2018 [9]	-	Normalization, 2D median filter, Otsu thresholding to identify background, background subtraction, adaptive thresholding to binarize, skeletonization, artifact removal, skeleton smoothing, identification of disconnected spines, geodesic distance transform from dendrite to identify spine seed locations, local geodesic distance transforms to extract features, neural network-based spine identification.	3D	Training data is open source. Does not provide full segmentation.	Yes
Singh et al., 2017 [10]	-	3D Gaussian filtering, binary thresholding of Hessian matrix, centerline extraction, seeding via voxel coding algorithm, thresholding of spine voxels.	3D	-	Yes, but not all processing steps worked in our hands.
On et al., 2017 [11]	DendritePA	Top hat filter, 2D median filter, image enhancement, segmentation using Otsu method, dendrite segmentation via piecewise convolution kernel method with low-pass filter, spine segmentation via distance map and subsequent watershed segmentation, spine categorization.	3D + time	Approach was compared to NeuronIQ. Can also perform fluorescence-based analyses of spines (e.g. co-localization of cofilin).	Yes
Xie et al., 2017 [12]	-	Image normalization, enhance linear structures, binary thresholding, skeletonization and branch point identification, spines are identified using alternative points in the skeleton.	3D	Also detects boutons, can be utilized to identify synapses. Only performs localization, no segmentation.	Upon request
⚫<br>Dickstein et al., 2016 [13]	Neurolucida 360	Based on Wearne et al., 2005 and Rodriguez et al., 2008.	3D	-	Licensed software
Blumer et al., 2015 [14]	-	Training: compute synthetic fluorescence images based on EM data.calculate spine and dendrite probability maps based on the PCA of dendrite-orthogonal slices of synthetic images.<br><br>Method: generate 2D slices,	3D + time	Semi-automated approach. Utilized correlative light and electron microscopy (CLEM) data for benchmarking. CLEM data is no longer available. Specialized on CA1	No

		generate spine probability maps using 9 orientation-dependent probability PCA models, binary thresholding of probability map.<br><br>Time-series analysis: rigid registration, spine matching via distance and detection probability of all possible spine paths.		pyramidal neurons. Requires re-training if done with a different microscope and/or cell type.	
Shi et al., 2014 [15]	-	Image filtering, adaptive thresholding, morphological filtering, ray casting, wavelet transform.	3D	Semi-automated spine classification possible.	No
Rada et al., 2014 [16]	-	2 Methods: (1) Median filter, Hessian matrix calculation, dot enhancement filter, adaptive thresholding of the original image, “morphological thinning”, skeletonization (2) generate training set using SIFT features and manual labelling, train linear SVM on manually labelled SIFT feature vectors. Spines in both methods are then segmented using watershed segmentation followed by a variational-based algorithm.	2D	Approach was compared to NeuronIQ.	No
Su et al., 2014 [17]	-	Median filter, gradient magnitude image normalization, custom Hessian filtering, white top-hat filtering to remove spines, binarization, skeletonization, dendrite boundary detection, dendrite subtraction, watershed segmentation of spines.	2D	Requires super-resolution images as input. Approach was compared to Zhang et al., 2007 and NeuronStudio. Can perform spine density calculations.	No
Ortiz et al., 2014 [18]	-	Method details unclear but based on simple linear iterative clustering resulting in supervoxels, which are then clustered using maximal inscribed spheres to segment spines.	3D	-	No
Erdil et al., 2012 [19]	-	Median filtering, edge-preserving smoothing, Otsu thresholding, extended maxima transform and boundary extraction, watershed segmentation, graph-theoretic segmentation and k-means clustering for final segmentation of spines.	2D	-	No

He et al., 2012 [20]	-	‘Regularized morphological filtering’, Otsu thresholding to binarize, non-linear degeneration equation to detect spines, thresholding to segment, spine classification.	3D	Approach was compared to Rodriguez et al., 2008, Zhang et al., 2009, and Fan et al., 2009. Can perform spine classification.	No
Swanger et al., 2011 [21]	-	Semi-automated dendrite detection based on thresholds in the IMARIS software suite.	3D + time	Time-course data analysis is possible but it is not automated; method implemented in IMARIS.	No
Son et al., 2011 [22]	-	Unsharp mask filtering, binarization, anisotropic diffusion filtering followed by ISODATA algorithm to obtain skeleton of dendrites and spines, end points are detected and used as seed points for geodesic active contour modelling of spines, watershed segmentation. Spine matching across time based on Lucas-Kanade method (optical flow of spine within predetermined region of another spine at the previous time point).	2D + time	Approach was compared to NeuronIQ. Offers automatic spine classification.	No
Mukai et al., 2011 [23]	Spiso-3D	Deconvolution, scale-space transformation, dendrite ridge line detection and subsequent 3D dendrite reconstruction, 3D dendrite subtraction and subsequent spine point determination via eigenvalues of the Hessian tensor, spine diameter calculation, manual correction.	3D	Semi-automated approach. Only performs localization, spine neck length and spine diameter calculations, no full segmentation.	No
Zhang et al., 2010 [24]	-	Iterative deconvolution, Otsu thresholding, gradient vector field calculation, feature point detection, spine detection via eigen-analysis method, segmentation via fast marching method, postprocessing.	3D	Intended to be used on medium-sized spiny neurons.	No
Li et al., 2010 [25]	-	Spine detection: Hessian matrix, curvilinear structure detection to identify dendrite, length-based criterion for spines and dendrites<br><br>Registration: iterative closest reciprocal point algorithm-based rigid transformation followed by	2D + time	This approach focuses on chronic data quantification. Approach was compared to Li et al., 2009, Koh et al., 2002, and Fan et al., 2009. First time an approach deals with dis- and	No

		non-rigid local deformation for fine-scale registration.<br><br>Spine matching: distance-based criterion, exclusive matching between time points, global similarity metric.		re-appearing spines. Dataset available upon request.	
Fan et al., 2009 [26]	-	Curvilinear dendrite detection, adaptive local binary fitting Level Set Model, Laplacian of Gaussian, Maximum Likelihood Estimation.	3D + time	Time-course data analysis possible.	Upon request
⚫ Yuan et al., 2009 [27]	Part of the FARSIGHT toolkit	Deconvolution, grayscale skeletonization, high-curvature and critical point detection, iterative path-line formation algorithm, Graph Generation & Computation of the Intensity-weighted Minimal Spanning Tree (IW-IMST), MDL-Based Estimation of Dendritic Backbones and spines.	3D	Evaluated on data from multiple laboratories. Informative section on related literature.	Available upon request, available open-source FARSIGHT toolkit
Li et al., 2009 [28]	-	3D median filter, top-hat filter, Rayburst sampling using user-defined seed points, fuzzy C-mean clustering, iterative low-pass filtering and mesh decimation, spine detection via region growing followed by watershed segmentation.	3D	Semi-automated. Approach was compared to NeuronStudio. Claims to be suitable for images containing multiple neurons/dendrites.	No
Janoos et al., 2009 [29]	-	Deconvolution, non-linear diffusion filtering, binarization, set distance boundaries and reconnect floating spine heads using active contour shape models, extended marching cubes algorithm to surface dendrites and spines, skeleton extraction using a medial geodesic function, spine detection using length-based branching criterion on skeleton.	3D	-	No
⚫⚫ Rodriguez et al., 2008 [30]	NeuronStudio	Adaptive local thresholding, voxel clustering and Rayburst Sampling.	3D	-	No longer available
Zhang et al., 2007 [31]	-	Curvilinear structure detection, linear discriminate analysis.	2D	-	No
⚫ Cheng et al., 2007 [32]	NeuronIQ	adaptive thresholding, backbone extraction, local-region-cutting algorithm, SNR-based threshold.	3D	-	No longer available

Bai et al., 2007 [33]	-	Deconvolution, unsharp mask filtering, 3D median filtering, binarization, size-dependent connected component analysis to determine dendrites and spines, skeletonization, angle- and width-based criterion on skeleton to identify and segment dendritic spines.	2D	Can also compute spine length.	No longer available
⚫⚫ Xu et al., 2006 [34]	NeuronIQ	Median filter, deconvolution, unsharp masking and subsequent binarization, medial axis transform to identify the skeleton, angle- and length-based exclusion of spine segments of the skeleton, grassfire algorithm to detect potential spine seed points, second grassfire algorithm to detect dendrite boundary.	3D	-	No
⚫⚫ Wearne et al., 2005 [35]	Part of the NeuronStudio package	Deconvolution, thresholding, skeletonization and subsequent medial axis calculation, Rayburst sampling, iterative thinning algorithm, custom cleanup of dendritic tree skeleton.	3D	Primarily utilized for neural morphology analysis. Basis for Rodriguez et al., 2008.	Licensed software
⚫ Weaver et al., 2004 [36]	3DMA-neuron	Deconvolution, 2x voxel compression in z, semi-automated data registration, manual tiling, binarization, skeletonization, dendrite radius estimation, spine detection using protrusions of spines from the dendrite as potential seed points, manual revision option at end.	3D + time	Further refined Koh et al., 2002 to extend use cases to large datasets (e.g. entire neuron data).	No longer available
⚫⚫ Koh et al., 2002 [37]	3DMA-neuron	Deconvolution, dendritic backbone extraction via medial axis algorithm. Active contour model, volume-based matching, spine categorization.	3D + time	Relatively slow due to computational burden.	No longer available
⚫⚫ Koh and Lindquist, 2001 [38]	3DMA-neuron	Deconvolution, dendritic backbone extraction via medial axis algorithm. Active contour model, volume-based matching, spine categorization.	3D + time	Relatively slow due to computational burden.	No longer available
⚫⚫ Watzel et al., 1995 [39]	-	Binarization, skeletonization, dendrite reconstruction and subsequent subtraction from binary image, generation of spine seeds by finding skeletal points adjacent to	3D	One of the first efforts to automate spine detection. Only works with one unbranched dendrite with all	No

		dendrite, seed classification, minimal box placement around each spine.		spines of interest connected to the dendrite.	
⚫ Rusakov and Stewart, 1995 [40]	-	Region of interest selection, binarization via valley global minimum detection, skeletonization, break bifurcation points and measure segments, manual classification of segments into spine, dendrite and “ignored”.	2D	One of the first efforts to automate spine detection. Semi-automated approach. Can perform spine density estimation.	No

Table A. Previous approaches of (semi)-automated spine detection.
⚫⚫ indicate landmark papers in the field. ⚫ denote significant contributions to the field. N.B. importance to the field was determined subjectively by the authors.

Dataset	Species	Mode	Acquisition technique	Dye/ Fluorophore	XY resolution [µm]	Z step [µm]
DeepD3 Benchmark	Rat	Ex vivo	Two-photon	tdTomato	0.094	0.50
External A	Mouse	In vivo	Two-photon	iGluSnFR	0.200	0.75
External B	Mouse	In vivo	Two-photon	Thy1-YFP	0.117	0.50
External C	Human	In vitro	Confocal	Biocytin + HRP + DAB	0.240	0.42

Table B. Datasets that were evaluated in this study. Data of External A was described in Kazemipour et al., 2019 [41]. Data of External B was described in Frank et al., 2018 [42]. Data of External C was described in Peng et al., 2015 [43] and Manubens-Gil et al., 2022 [44]. HRP = Horseradish peroxidase, DAB = 3,3'-Diaminobenyidine tetrahydrochloride.

Model	f_{base}	Trained on	Output
DeepD3_8F_94nm.h5	8	Complete training data, but rescaled to match an xy-resolution of 94 nm	Spine and dendrite prediction maps
DeepD3_16F_94nm.h5	16		
DeepD3_32F_94nm.h5	32		
DeepD3_8F.h5	8	Complete training data with various xy-resolutions	
DeepD3_16F.h5	16		
DeepD3_32F.h5	32		

Table C. Model Zoo. All models were trained on the DeepD3 training dataset using the different f_{base} configurations. We offer two sets of models: trained with original resolution and trained on rescaled data to match a 94 nm resolution. Each network yield two prediction maps: dendrites and dendritic spines with a probability between 0 (px does not belong to class) and 1 (px belongs certainly to class).

References

1. Ekaterina, P., Peter, V., Smirnova, D., Vyacheslav, C., & Ilya, B. (2023). SpineTool is an open-source software for analysis of morphology of dendritic spines. *Scientific Reports*, 13(1), 10561.
2. Vogel, F. W., Alipek, S., Eppler, J. B., Triesch, J., Bissen, D., Acker-Palmer, A., ... & Kaschube, M. (2023). Fully automated detection of dendritic spines in 3D live cell imaging data using deep convolutional neural networks. bioRxiv, 2023-01.
3. Argunşah, A. Ö., Erdil, E., Ghani, M. U., Ramiro-Cortés, Y., Hobbiss, A. F., Karayannis, T., ... & Ünay, D. (2022). An interactive time series image analysis software for dendritic spines. *Scientific Reports*, 12(1), 12405.
4. Vidaurre-Gallart, I., Fernaud-Espinosa, I., Cosmin-Toader, N., Talavera-Martınez, L., Martin-Abadal, M., Benavides-Piccione, R., Gonzalez-Cid, Y., Pastor, L., DeFelipe, J., & Garcıa-Lorenzo, M. (2022). A deep learning-based workflow for dendritic spine segmentation. *Frontiers in neuroanatomy*, *16*, 817903–817903.
5. Das, N. et al. 3dspan: An interactive software for 3d segmentation and analysis of dendritic spines. Neuroinformatics 1–20540 (2021).
6. Levet, F., Tønnesen, J., Nägerl, U. V. & Sibarita, J.-B. Spinej: a software tool for quantitative analysis of nanoscale spine morphology. Methods 174, 49–55 (2020).
7. Rada, L., Kilic, B., Erdil, E., Ramiro-Cortés, Y., Israely, I., Unay, D., ... & Argunsah, A. Ö. (2018). Tracking-assisted detection of dendritic spines in time-lapse microscopic images. Neuroscience, 394, 189-205.
8. Xiao, X., Djurisic, M., Hoogi, A., Sapp, R. W., Shatz, C. J., & Rubin, D. L. (2018). Automated dendritic spine detection using convolutional neural networks on maximum intensity projected microscopic volumes. *Journal of neuroscience methods*, *309*, 25–34.
9. Smirnov, M. S., Garrett, T. R., & Yasuda, R. (2018). An open-source tool for analysis and automatic identification of dendritic spines using machine learning. *Plos one*, *13*(7), e0199589.
10. Singh, P. K., Hernandez-Herrera, P., Labate, D., & Papadakis, M. (2017). Automated 3-d detection of dendritic spines from in vivo two-photon image stacks. *Neuroinformatics*, *15*(4), 303–319.
11. On, V., Zahedi, A., Ethell, I. M., & Bhanu, B. (2017). Automated spatiotemporal analysis of dendritic spines and related protein dynamics. *Plos one*, *12*(8), e0182958.
12. Xie, Q., Chen, X., Deng, H., Liu, D., Sun, Y., Zhou, X., Yang, Y., & Han, H. (2017). An automated pipeline for bouton, spine, and synapse detection of in vivo two-photon images. *BioData Mining*, *10*(1), 1–23.
13. Dickstein, D. L., Dickstein, D. R., Janssen, W. G., Hof, P. R., Glaser, J. R., Rodriguez, A., O'Connor, N., Angstman, P., & Tappan, S. J. (2016). Automatic dendritic spine quantification from confocal data with neurolucida 360. *Current protocols in neuroscience*, *77*(1), 1–27.
14. Blumer, C., Vivien, C., Genoud, C., Perez-Alvarez, A., Wiegert, J. S., Vetter, T., & Oertner, T. G. (2015). Automated analysis of spine dynamics on live ca1 pyramidal cells. *Medical image analysis*, *19*(1), 87–97.
15. Shi, P., Huang, Y., & Hong, J. (2014). Automated three-dimensional reconstruction and morphological analysis of dendritic spines based on semisupervised learning. *Biomedical optics express*, *5*(5), 1541–1553.
16. Rada, L., Erdil, E., Argunsah, A. O., Unay, D., & Cetin, M. (2014). Automatic dendritic spine detection using multiscale dot enhancement filters and sift features. *2014 IEEE International Conference on Image Processing (ICIP)*, 26–30.
17. Su, R., Sun, C., Zhang, C., & Pham, T. D. (2014). A novel method for dendritic spines detection based on directional morphological filter and shortest path. *Computerized Medical Imaging and Graphics*, *38*(8), 793–802.
18. Ortiz, C. A., Gonzalo-Martı, C., Pen˜a, J. M., & Menasalvas, E. (2014). 3d dendrite spine detection-a supervoxel based approach. In *Rough sets and intelligent systems paradigms* (pp. 359–366). Springer.
19. Erdil, E., Yagci, A. M., Argunsah, A. O., Ramiro-Cort´es, Y., Hobbiss, A. F.,¨ Israely, I., & Unay, D. (2012). A tool for automatic dendritic spine detection and analysis. part i: Dendritic spine detection using multilevel region-based segmentation. *2012 3rd International Conference on Image Processing Theory, Tools and Applications (IPTA)*, 167–171.
20. He, T., Xue, Z., & Wong, S. T. (2012). A novel approach for three dimensional dendrite spine segmentation and classification. *Medical Imaging 2012: Image Processing*, *8314*, 919–926.
21. Swanger, S. A., Yao, X., Gross, C., & Bassell, G. J. (2011). Automated 4d analysis of dendritic spine morphology: Applications to stimulus-induced spine remodeling and pharmacological rescue in a disease model. *Molecular brain*, *4*(1), 1–14.

22. Son, J., Song, S., Lee, S., Chang, S., & Kim, M. (2011). Morphological change tracking of dendritic spines based on structural features. *Journal of microscopy,* 241(3), 261-272.
23. Mukai, H., Hatanaka, Y., Mitsuhashi, K., Hojo, Y., Komatsuzaki, Y., Sato, R., Murakami, G., Kimoto, T., & Kawato, S. (2011). Automated analysis of spines from confocal laser microscopy images: Application to the discrimination of androgen and estrogen effects on spinogenesis. *Cerebral cortex*, *21*(12), 2704–2711.
24. Zhang, Y., Chen, K., Baron, M., Teylan, M. A., Kim, Y., Song, Z., Greengard, P., & Wong, S. T. (2010). A neurocomputational method for fully automated 3d dendritic spine detection and segmentation of medium-sized spiny neurons. *Neuroimage*, *50*(4), 1472–1484.
25. Li, Q., Deng, Z., Zhang, Y., Zhou, X., Nagerl, U. V., & Wong, S. T. (2010). A global spatial similarity optimization scheme to track large numbers of dendritic spines in time-lapse confocal microscopy. *IEEE Transactions on Medical Imaging*, *30*(3), 632–641.
26. Fan, J., Zhou, X., Dy, J. G., Zhang, Y., & Wong, S. T. (2009). An automated pipeline for dendrite spine detection and tracking of 3d optical microscopy neuron images of in vivo mouse models. *Neuroinformatics*, *7*(2), 113–130.
27. Yuan, X., Trachtenberg, J. T., Potter, S. M., & Roysam, B. (2009). Mdl constrained 3-d grayscale skeletonization algorithm for automated extraction of dendrites and spines from fluorescence confocal images. *Neuroinformatics*, *7*(4), 213–232.
28. Li, Q., Zhou, X., Deng, Z., Baron, M., Teylan, M. A., Kim, Y., & Wong, S. T. (2009). A novel surface-based geometric approach for 3d dendritic spine detection from multi-photon excitation microscopy images. *2009 IEEE International Symposium on Biomedical Imaging: From Nano to Macro*, 1255–1258.
29. Janoos, F., Mosaliganti, K., Xu, X., Machiraju, R., Huang, K., & Wong, S. T. (2009). Robust 3d reconstruction and identification of dendritic spines from optical microscopy imaging. *Medical image analysis*, *13*(1), 167– 179.
30. Rodriguez, A., Ehlenberger, D. B., Dickstein, D. L., Hof, P. R., & Wearne, S. L. (2008). Automated three-dimensional detection and shape classification of dendritic spines from fluorescence microscopy images. *PloS one*, *3*(4), e1997.
31. Zhang, Y., Zhou, X., Witt, R. M., Sabatini, B. L., Adjeroh, D., & Wong, S. T. (2007). Dendritic spine detection using curvilinear structure detector and lda classifier. *Neuroimage*, *36*(2), 346–360.
32. Cheng, J., Zhou, X., Miller, E., Witt, R. M., Zhu, J., Sabatini, B. L., & Wong, S. T. (2007). A novel computational approach for automatic dendrite spines detection in two-photon laser scan microscopy. *Journal of neuroscience methods*, *165*(1), 122–134.
33. Bai, W., Zhou, X., Ji, L., Cheng, J., & Wong, S. T. (2007). Automatic dendritic spine analysis in two-photon laser scanning microscopy images. *Cytometry Part A: The Journal of the International Society for Analytical Cytology*, *71*(10), 818–826.
34. Xu, X., Cheng, J., Witt, R. M., Sabatini, B. L., & Wong, S. T. (2006). A shape analysis method to detect dendritic spine in 3d optical microscopy image. *3rd IEEE International Symposium on Biomedical Imaging: Nano to Macro, 2006.*, 554–557.
35. Wearne, S., Rodriguez, A., Ehlenberger, D., Rocher, A., Henderson, S., & Hof, P. (2005). New techniques for imaging, digitization and analysis of three-dimensional neural morphology on multiple scales. *Neuroscience*, *136*(3), 661–680.
36. Weaver, C. M., Hof, P. R., Wearne, S. L., & Lindquist, W. B. (2004). Automated algorithms for multiscale morphometry of neuronal dendrites. *Neural computation*, *16*(7), 1353–1383.
37. Koh, I. Y., Lindquist, W. B., Zito, K., Nimchinsky, E. A., & Svoboda, K. (2002). An image analysis algorithm for dendritic spines. *Neural computation*, *14*(6), 1283–1310.
38. Koh, I. Y., & Lindquist, W. B. (2001). Automated 3d dendritic spine detection and analysis from two-photon microscopy. *Three-Dimensional and Multidimensional Microscopy: Image Acquisition and Processing VIII*, *4261*, 48–59.
39. Watzel, R., Braun, K., Hess, A., Scheich, H., & Zuschratter, W. (1995). Detection of dendritic spines in 3-dimensional images. In *Mustererkennung 1995* (pp. 160–167). Springer.
40. Rusakov, D. A., & Stewart, M. G. (1995). Quantification of dendritic spine populations using image analysis and a tilting disector. *Journal of neuroscience methods*, *60*(1-2), 11–21.
41. Kazemipour, A., Novak, O., Flickinger, D., Marvin, J. S., Abdelfattah, A. S., King, J., Borden, P. M., Kim, J. J., Al-Abdullatif, S. H., Deal, P. E., et al. (2019). Kilohertz frame-rate two-photon tomography. *Nature methods*, 16(8), 778–786.
42. Frank, A. C., Huang, S., Zhou, M., Gdalyahu, A., Kastellakis, G., Silva, T. K., Lu, E., Wen, X., Poirazi, P., Trachtenberg, J. T., et al. (2018). Hotspots of dendritic spine turnover facilitate clustered spine addition and learning and memory. *Nature communications*, 9(1), 422.
43. Peng, H., Hawrylycz, M., Roskams, J., Hill, S., Spruston, N., Meijering, E., & Ascoli, G. A. (2015). Bigneuron: Large-scale 3d neuron reconstruction from optical microscopy images. *Neuron*, 87(2), 252–256.

44. Manubens-Gil, L., Zhou, Z., Chen, H., Ramanathan, A., Liu, X., Liu, Y., ... & Peng, H. (2023). BigNeuron: a resource to benchmark and predict performance of algorithms for automated tracing of neurons in light microscopy datasets. *Nature Methods*, 20, 824–835
